# Biomechanical evaluation of percutaneous compression plate and femoral neck system in Pauwels type III femoral neck fractures

**DOI:** 10.1186/s10195-024-00792-0

**Published:** 2024-11-29

**Authors:** Xiaoping Xie, Songqi Bi, Qingxu Song, Qiong Zhang, Zhixing Yan, Xiaoyang Zhou, Tiecheng Yu

**Affiliations:** Department of Orthopedics, Orthopedics Center, First Hospital of Jilin University, Jilin University, Changchun, China

**Keywords:** Inverted cannulated compression screw, Femoral neck system, Percutaneous compression plate, Biomechanics, Femoral neck fractures

## Abstract

**Background:**

The optimal treatment for Pauwels type III femoral neck fractures remains contentious. We aim to compare the biomechanical properties of three inverted cannulated compression screw (ICCS), femoral neck system (FNS), and percutaneous compression plate (PCCP) to determine which offers superior stability for unstable femoral neck fractures.

**Materials and methods:**

Finite element analysis and artificial bone models were used to establish Pauwels III femoral neck fracture models. They were divided into ICCS, FNS, and PCCP groups based on respective internal fixation assemblies. The models were subjected to vertical axial loads (2100 N) and torsional forces (10 N × mm) along the femoral neck axis in the finite element analysis. The primary outcomes such as the *Z* axis fragmentary displacements, as well as displacements and the von Mises stress (VMS) distributions of internal fixations, were analyzed. Additionally, the artificial bones were subjected to progressively increasing vertical axial pressures and torsional moments at angles of 2°, 4°, and 6°, respectively. The vertical displacements of femoral heads and the required torque values were recorded.

**Results:**

Finite element analysis revealed that under single-leg stance loading, the maximum *Z*-axis fragmentary displacements were 5.060 mm for ICCS, 4.028 mm for FNS, and 2.796 mm for PCCP. The maximum displacements of internal fixations were 4.545 mm for ICCS, 3.047 mm for FNS, and 2.559 mm for PCCP. Peak VMS values were 512.21 MPa for ICCS, 242.86 MPa for FNS, and 413.85 MPa for PCCP. Under increasing vertical loads applied to the artificial bones, the average vertical axial stiffness for the ICCS, FNS, and PCCP groups were 244.86 ± 2.84 N/mm, 415.03 ± 27.10 N/mm, and 529.98 ± 23.08 N/mm. For the torsional moment tests, the PCCP group demonstrated significantly higher torque values at 2°, 4°, and 6° compared with FNS and ICCS, with no significant difference between FNS and ICCS (*P* > 0.05).

**Conclusions:**

Finite element analysis and artificial bone models indicated that PCCP offers the best compressive and rotational stability for fixing Pauwels type III femoral neck fractures, followed by FNS and then ICCS. No significant difference in rotational resistance was observed between FNS and ICCS in synthetic bones.

*Level of Evidence* Level 5.

**Supplementary Information:**

The online version contains supplementary material available at 10.1186/s10195-024-00792-0.

## Introduction

Femoral neck fractures are prevalent concerns in clinical practice, frequently stemming from falls, trauma, and vehicular accidents, among other causes. With the aging of the population, the incidence of hip fracture is notably increasing. It has been projected that the global count of hip fracture patients is set to rise to 2.6 million by 2025 and is expected to surge to 4.5 million by 2050 [1], with femoral neck fractures constituting approximately 50% of these cases [2]. Femoral neck fractures are predominantly seen in the elderly, primarily due to low-energy falls. In contrast, these fractures are relatively rare in healthy young adults, where they usually result from high-energy trauma [3, 4].

Displaced femoral neck fractures in elderly patients are commonly managed with joint arthroplasty [5]. However, this approach may not be optimal for younger patients (under 60 years of age) with femoral neck fractures, as the longevity of the arthroplasty may not be as satisfactory compared with its application in older, lower-demand patients. When such fractures occur in younger individuals, they often feature a more vertical fracture line, as indicated by a high Pauwels angle [6]. This vertical orientation increases shear forces at the fracture site, which can lead to complications such as hip varus, collapse, nonunion, avascular necrosis of the femoral head, or internal fixation failure [7, 8]. Consequently, the management of femoral neck fractures in the young adults faced numerous challenges in orthopedic trauma surgery [9]. Timely anatomic reduction and stable internal fixation are essential to mitigate distortion of proximal femoral vessels, preserve femoral head blood supply, and create a suitable biomechanical environment for fracture healing [10, 11]. Operative intervention is generally preferred for managing the majority of femoral neck fractures.

Over the years, there has been an ongoing debate about the optimal internal fixation operation for young patients with femoral neck fractures, including surgical timing, open versus closed reduction, surgical approach, and fixation methods. With the continuous development and advancement of medical technology, internal fixation devices undergo constant innovation. Regardless of the treatment method, the aim remains to achieve anatomical reduction, ensure stable internal fixation, and minimize the occurrence of complications [12].

Three inverted cannulated compression screws (ICCS) were generally accepted treatment methods for nondisplaced femoral neck fractures (Garden I and II), with the aim of preventing fracture displacement [13–15]. These screws offered advantages such as minimally invasive procedures, easy operation, less tissue damage, and dynamic compression [12, 16].However, ICCS may lose the advantage of dynamic compression in patients with unstable Pauwels III femoral neck fractures, potentially increasing the risk of nonunion and avascular necrosis of the femoral head after surgery [7]. In addition, these patients were often required to limit weight-bearing exercises for 6 to 12 weeks postsurgery until sufficient healing progress allowed weight bearing [16]. Therefore, stable fixation is crucial for reducing the incidence of common postoperative complications, such as nonunion, avascular necrosis of the femoral head, fixation failure, and femoral neck shortening, which could potentially impact long-term outcomes [17]. 

In recent years, two internal fixation devices have been introduced for the treatment of femoral neck fractures: percutaneous compression plate (PCCP) and femoral neck system (FNS). Both devices have demonstrated improved clinical efficacy and reduced postoperative complications [18–21]. However, there is limited research on the biomechanical properties of PCCP and FNS.

This study aims to compare the biomechanical properties of PCCP, FNS, and ICCS fixation for Pauwels type III femoral neck fractures using finite element analysis and artificial bone models. The primary outcomes included the *Z*-axis fragmentary displacements, as well as the displacements and von Mises stress (VMS) of internal fixation devices in the finite analysis and axial stiffness and torsional moment in artificial bone models, while secondary outcomes included the torsional displacements of fracture surfaces. Stable internal fixation may reduce the incidence of postoperative complications and improve long-term functional outcomes. These findings will potentially guide surgeons toward more effective treatment strategies for femoral neck fractures.

## Material and methods

**Fig. 1:**
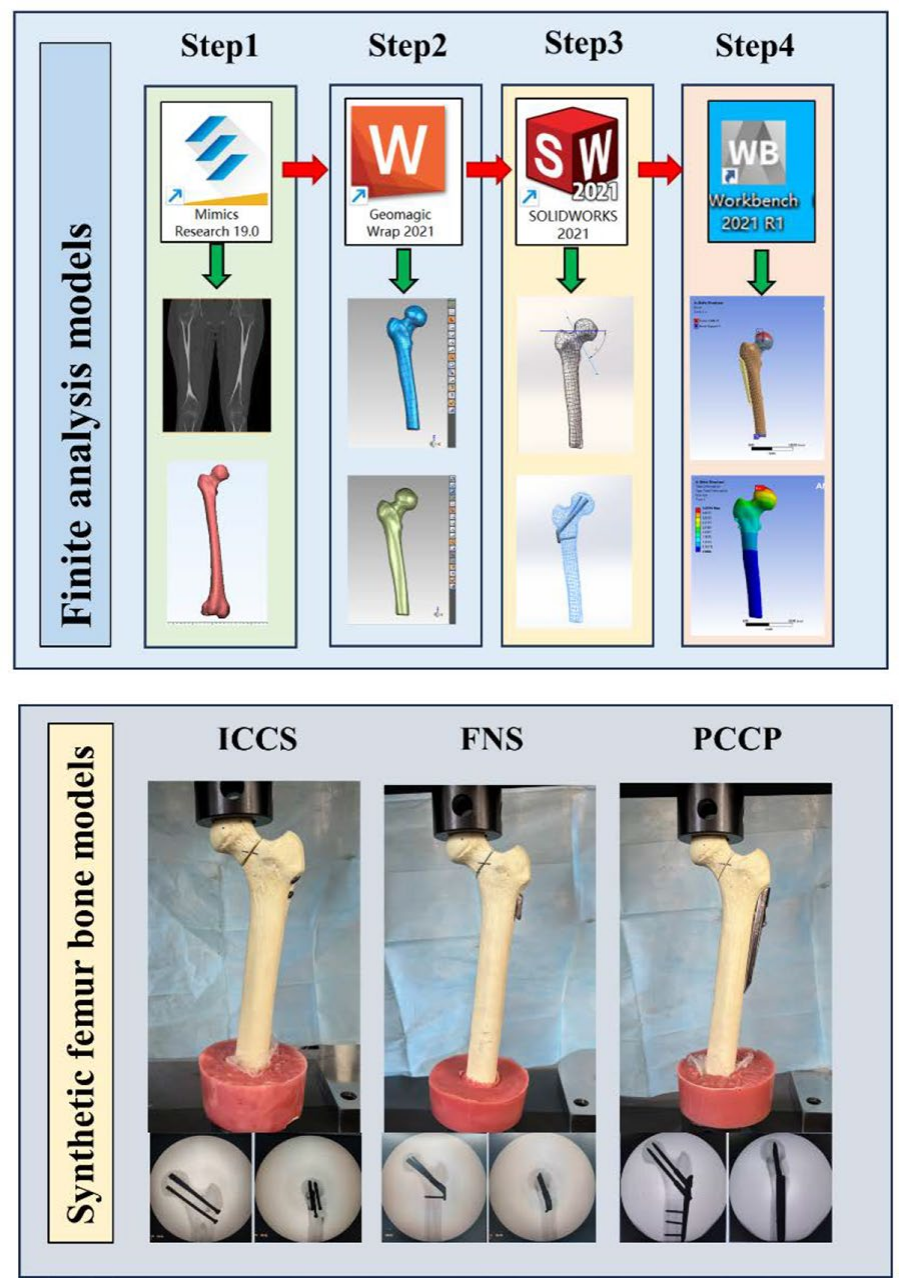
The schematic diagram of finite element analysis in this study

The study conducts finite element analysis to assess the displacement of the femur and internal fixation, rotational displacement of the fracture site, and stress on internal fixation under different fixation methods. Subsequently, artificial bone models were used to evaluate the compressive strength, maximum load-bearing capacity, and rotational stability under different fixation methods. The schematic diagram of research workflow is depicted in the Fig. 1.

### Three-dimensional modeling of the femur

The requirement for informed consent was waived. This study was approved by the Ethics Committee of the First Hospital of Jilin university. A healthy 30-year-old female volunteer was selected, and her femur was scanned using a 64-slice computed tomography (CT) scanner (Siemens, Germany). The CT data, stored in DICOM format, was imported into software Mimics 21.0 (Materialise, Belgium) for the three-dimensional (3D) reconstruction. Subsequently, the 3D model of the upper femur was extracted based on tissue grayscale values and region segmentation and exported in stereolithography (STL) format. The STL models underwent smoothing, meshing, noise reduction, and surface fitting in Geomagic Wrap 2021 (Geomagic, USA) before being processed in SolidWorks 2021 (Dassault, France) to construct the proximal femoral bone models of cortical and cancellous bone using Boolean operations and reassembly.

### Fracture models

**Fig. 2:**
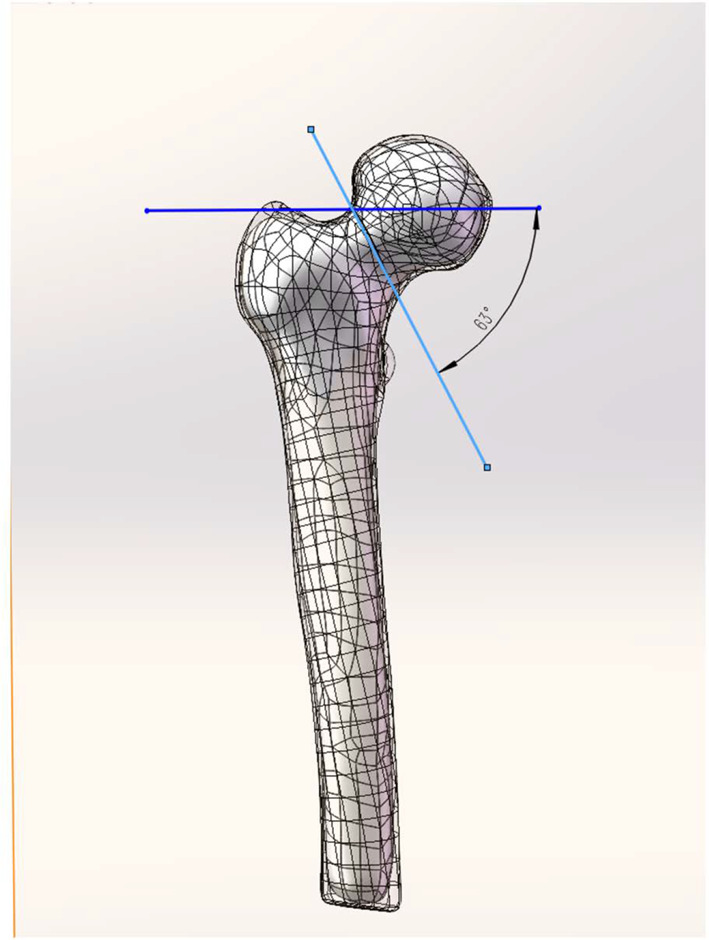
Pauwels III femoral neck fracture model with Pauwels angles of 70°. A mean varus angle of 7° between the anatomical axis of the femur and the line of force of the lower limb was considered. Therefore, the fracture line was chosen to be at a 63° angle to the neutral position of the femur

The femoral neck fracture models were established according to the Pauwels classification by creating a virtual osteotomy in SolidWorks 2021 [22]. First, we established a horizontal line through the center of the femoral head. Given the average varus angle of 7° between the anatomical axis of the femur and the line of force of the lower limb, the fracture line was selected to be at an angle of 63° relative to the horizontal line under the neutral position of the femurs (Fig. 2).

### The internal fixation models

According to the geometric characteristics of these internal fixation devices, the models of ICCS, FNS, and PCCP were generated in SolidWorks 2021 (Supplementary Figs. 1–3). The ICCS model consisted of three 7.3-mm-diameter cannulated screws arranged in an inverted triangular configuration, aligned in parallel [20]. The tips of three independent screws were located 5 mm below the cortical bone of the femoral head. For the FNS model, the “nail-in-nail” design involved placing a 6.4 mm antirotation screw at an angle of 7.5°, which was then locked with a 10 mm sliding bolt, followed by the combination with 130° locking plate and fixation to the femoral shaft [23]. The tip of the sliding bolt was approximately 5 mm from the cortical bone of the femoral head. At the distal end of plate, one hole was made for a 5-mm-diameter locking screw. In the PCCP model, two 7.5-mm-diameter hip screws were positioned at an angle of 135° to the locking plate, with three locking screws at the distal end of locking plate [21]. The tips of two hip screws were also positioned 5 mm below the cortical bone of the femoral head. Since the focus of this study is unrelated to threads, the threads on the screws were modeled as smooth surfaces to simplify the model. The constructed fracture and internal fixation models were assembled and subsequently imported into the ANSYS Workbench software (ANSYS2021, USA) for finite element analysis.

### Finite element analysis

Each component of the models was meshed using solid tetrahedral elements. The material parameters for these models were assumed to be continuous, isotropic, and uniformly linear elastic. The PCCP was assigned a material of stainless steel, while the FNS and ICCS were assigned a material of titanium alloy (Ti-6Al-7Nb). The material parameters for each component are detailed in Table 1 [24, 25]. All models utilized tetrahedral mesh elements with a mesh size of 1 mm (Supplementary Fig. 4). The number of mesh elements ranged from 2,040,852 to 2,168,001, and the number of nodes ranged from 2,935,873 to 3,130,488 (Table 2). The average Jacobian factors were 1.0122 for ICCS, 1.0114 for FNS, and 1.0137 for PCCP. The average skewness values were 0.2279 for ICCS, 0.2281for FNS, and 0.2285 for PCCP. The current mesh density was deemed acceptable. In accordance with the well-established and approved contact method outlined in previous studies, the fracture surface was designated a friction contact with a coefficient of 0.46, and a binding contact was formed between the implant and femur models [25–27].

**Table 1:** Material parameters used in this study (titanium alloy, stainless steel, cortical, and cancellous)

Finite elements mode	Young’s modulus (Gpa)	Poisson’s ratio
Titanium alloy	105	0.35
Stainless steel (for PCCP)	195	0.3
Cortical bone	16.8	0.30
Cancellous bone	0.84	0.20

**Table 2:** The mesh information and quality of the finite element model

	ICCS	FNS	PCCP
Maximum size of element, mm	1	1	1
Number of nodes	2,935,873	2,942,467	3,130,488
Number of units	2,040,852	2,045,170	2,168,001
Jacobian ratio (mean ± SD)	1.0122 ± 0.0400	1.0114 ± 0.0585	1.0137 ± 0.0148
Skewness (mean ± SD)	0.2279 ± 0.1227	0.2281 ± 0.1227	0.2285 ± 0.1230

**Fig. 3:**
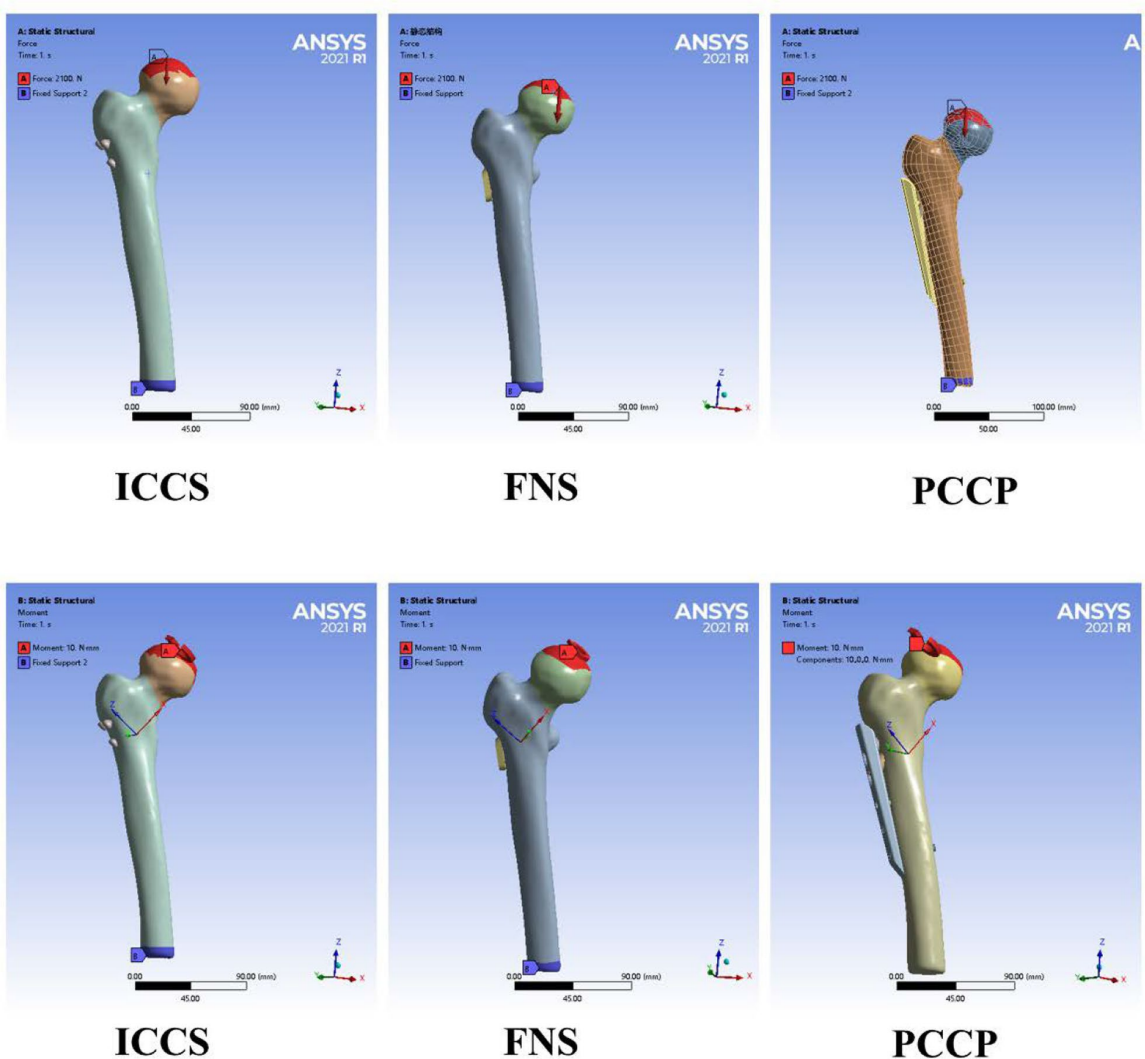
The femur was loaded in the single-leg stance in each finite element model. **a** Loads of 2100 N, equivalent to tripling the body weight of the subject, were vertically applied to the upper of the femoral head. **b** A torque force of 10 N × mm was applied along the axis of the femoral neck on the surface of the femoral head

For the calculations, the distal end of the femur was fully immobilized. Each assembly model extended angled 7° outward in alignment with findings from Van Houcke et al. [28]. It was determined that the force exerted on the joint during a single-leg stance was approximately three times the body weight. Consequently, the center of the femoral head was subjected an approximately vertical force of 2100 N (about 300% of 70 kg body weight) to simulate the load during walking [27, 29] (Fig. 3). Given that the impact of gravity on the results was considered negligible compared with the applied loads, it was excluded from the simulation to simplify the model and reduce computational complexity. This finite element model employed static simulations to evaluate biomechanical changes under loads, without considering preload conditions. Evaluation criteria included measuring the fracture displacements and stresses on the femur and internal fixations across different groups. Additionally, a torque force of 10 N × mm was applied along the axis of the femoral neck on the surface of the femoral head to assess the torsional displacements of the fracture plane (Fig. 3).

### Specimen preparation

**Fig. 4:**
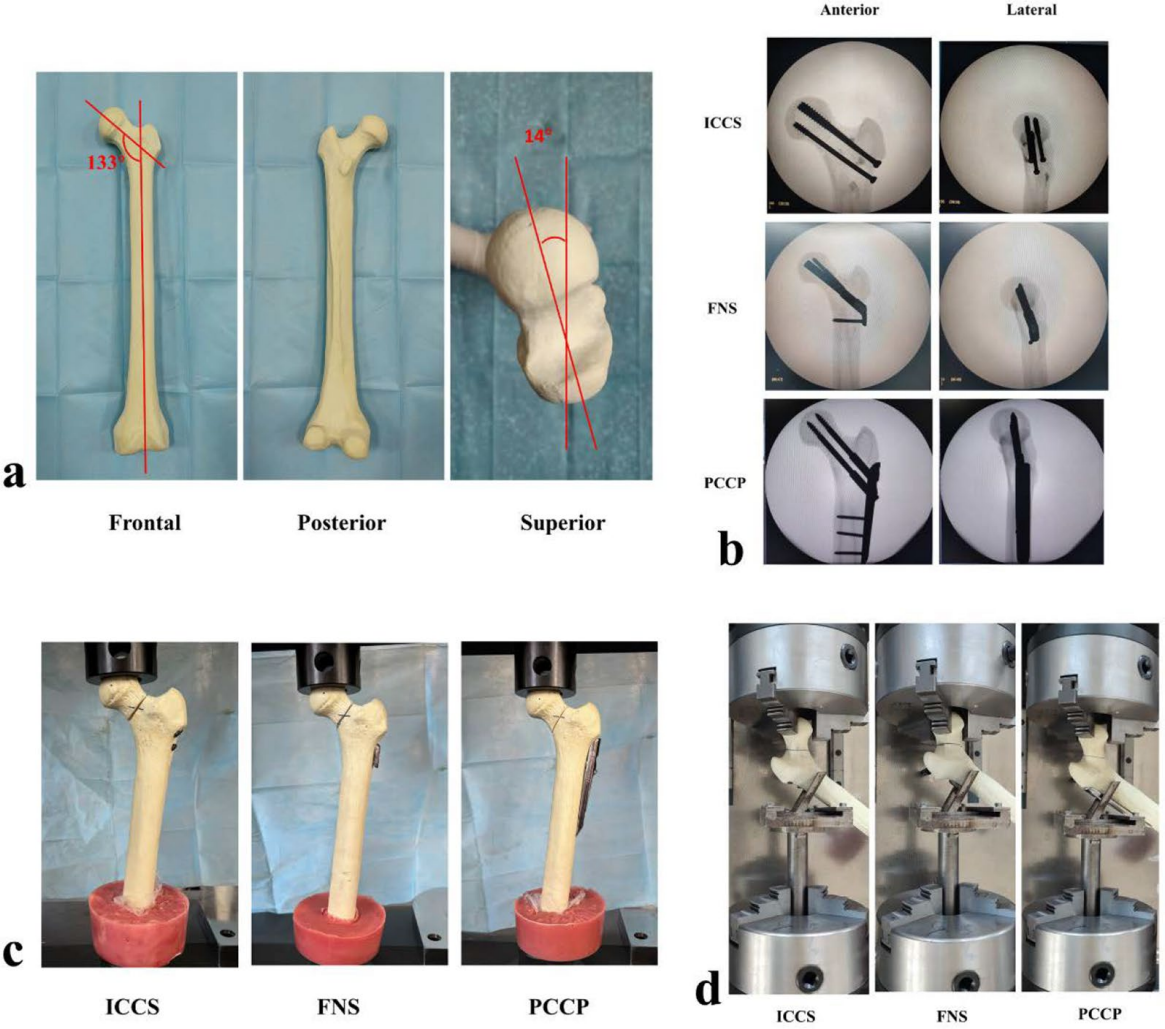
**a** Frontal, posterior, and superior views of a representative femur model, respectively. The neck-shaft angles of all synthesized femur models were 133°, and the femoral anteversion angles were 14°. **b** Anteroposterior and lateral views of three groups: ICCS, FNS, and PCCP. **c** Continuous load testing of Pauwels type III femoral neck fracture specimens under different internal fixation methods. **d** Torque testing of Pauwels type III femoral neck fracture specimens at various torsional angles

Eighteen identically shaped left side artificial bone models (ENOVO, China) were purchased (Fig. 4a) and randomly divided into the following three groups: PCCP, FNC, and ICCS. Polyurethane (PU) artificial bones are commonly utilized in biomechanical testing to mitigate the variability and limited availability of human bone specimens [30]. Artificial bones can be manufactured from PU using molds. A notable example of such artificial bones with a standardized geometry is Sawbone (Sawbone, Vashon Island, USA)[31, 32].

To ensure anatomical reduction and optimal implant placement, all models were predrilled for their respective fixations under the C-arm fluoroscopic guidance prior to osteotomy. Subsequently, the fixations were removed, and the Pauwels type III femoral neck fracture model was created by performing an osteotomy at an angle 63° relative to the horizontal plane. After the osteotomy, the fixations were reinserted.

### Surgical technique

The implantations were performed according to the manufacturers’ guidelines. The image of the internal fixation devices is shown in Supplementary Fig. 5. In the ICCS group, three guidewires were advanced through the femoral head, and their positions and angles were confirmed under X-ray fluoroscopy. The pilot holes were then drilled along the guidewire, followed by the parallel insertion of three 7.3 mm cannulated screws (100 mm inferior and 90 mm anterior and posterior in length; Wego company, China) into the prepared channels [33]. 

In the FNS group (DePuy Synthes, USA), a temporary guide K-wire was drilled for the prevention of femoral head rotation during the bolt insertion. The bolt was inserted 10% inferiorly to the femoral head center in the anterior–posterior direction and centrally in lateral direction via X-ray fluoroscopy monitoring.

The PCCP (Orthofix, USA) had an angulation of 135°. Similarly, a Kirschner wire was positioned to prevent the femoral head from rotation during the insertion of neck screws. The PCCP was installed using a plate mounting system by a consultant orthopedic surgeon. The lower femoral screw was placed just above the calcar in the anterior–posterior direction and centrally in lateral direction guided by X-ray fluoroscopy.

In all groups, the tips of neck screws extended to the subchondral bone of the femoral head, maintaining a tip-apex distance ranging from 5 to 10 mm. After implantation, radiographs in anterior–posterior and lateral views were taken to confirm the correct reduction of the fracture and accurate position of the implant devices (Fig. 4b).

### Biomechanical analysis

The distal end of each artificial bone model was resected a length of 20 cm and then affixed to the foundation base made by the denture powder. The models were oriented in a standing neutral position (without any adduction, abduction, flexion, or extension) and firmly placed on a CCS0-44100 electronic testing machine (Changchun Testing Machine Institute, Changchun, China). The upper part of the femoral head was in contact with the hemispherical mold to ensure the application of uniform force (Fig. 4c). Before initiating the measurements, a preloaded of 100 N was applied to each model to ensure full contact between the fragments.

Vertical loads (up to 1000 N) were applied to the femoral head. The displacements of the specimens were recorded at vertical loads of 200, 400, 600, 800, and 1000 N [34]. The slope of the load–displacement curve was interpreted as axial stiffness of the femoral head. Subsequently, these specimens were subjected to the ultimate load test, where vertical load levels were increased until the fixation system failed. Fixation failure was defined by one of the following criteria: (1) head screws cutting out, (3) fracture displacement exceeding 5 mm, or (3) failure of the implant devices.

For the torsion test, the proximal end of the femoral shaft was fixed on one side of the torsion testing machine using a custom-made fixture, with the femoral head affixed on the opposite side (Fig. 4d). The femoral neck axis was aligned with the machine torsion axis. The rotation direction was set to the frontal side of the femoral neck at a rate of 0.036° per minute. The torques of each group were recorded by the computer at the rotation angles of 2°, 4°, and 6°.

### Statistical analysis

Statistical analysis was conducted using SPSS software (version 25.0, IBM, USA). The data were shown as the mean ± standard deviation (SD). Significant differences among these groups were evaluated using one-way analysis of variance (ANOVA), followed by LSD post hoc multiple comparison tests. A *P* < 0.05 was considered statistically significant.

## Results

### Finite element analysis

#### Displacement of the femur

**Fig. 5:**
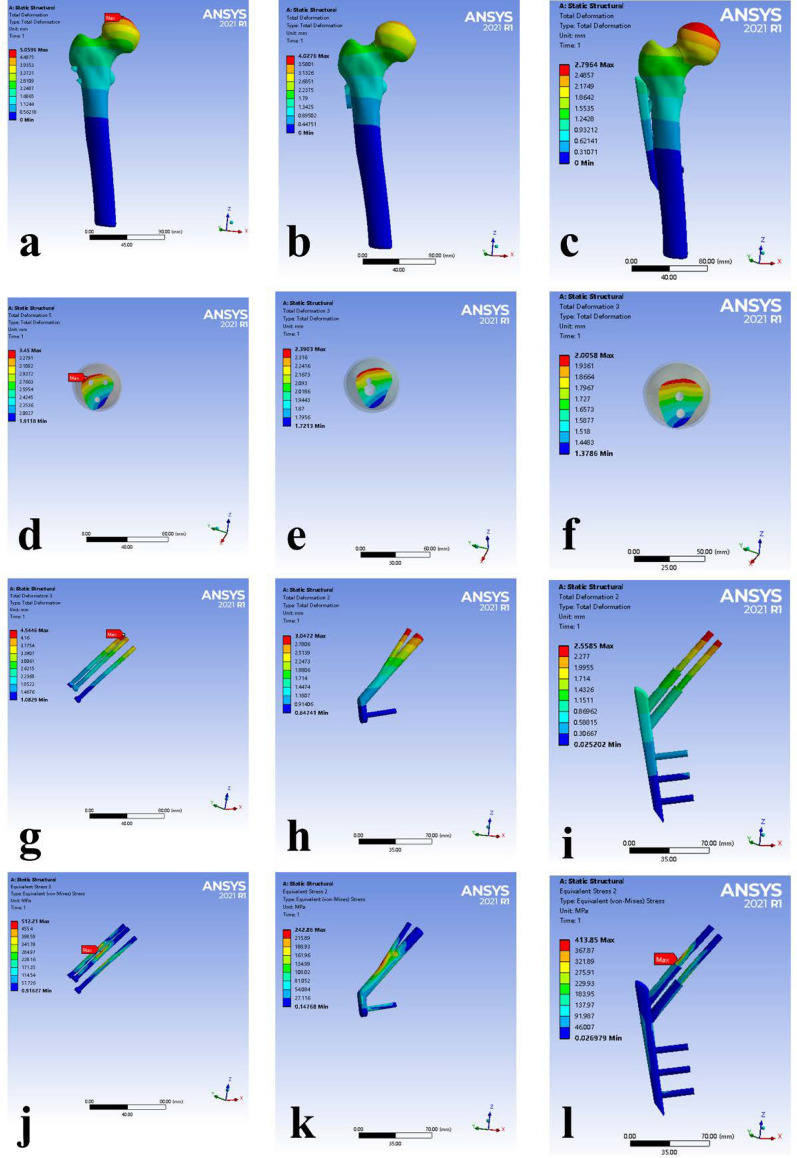
Biomechanical outcomes in the finite element analysis models for ICCS (**a**,** d**,**g**,** j**), FNS (**b**,**e**,** h**, **k**), and PCCP (**e**,**f**,** i**,** l**). (**a**–** c**) The displacements of the femur. (**d**–**f**) The maximum torsional displacements of the femur heads. (**g**–**i**) The displacement of the internal fixation (mm). (**j**–**l**) Von Mises stress (VMS) values of the internal fixation components.

The displacement contours of the femur indicated that the maximum displacement occurred at the upper portion of the femoral head. Specifically, the maximum femoral head displacements were 5.060 mm for the ICCS, 4.028 mm for the FNS, and 2.796 mm for the PCCP **(**Fig. 5a–c**)**. Furthermore, the maximum torsional displacements of fracture surfaces near the femoral head side were observed in the upper posterior part. The maximum torsional displacements of fracture surfaces were 3.45 mm for ICCS, 2.390 mm for FNS, and 2.006 mm for PCCP (Fig. 5d–f and Table 3).

**Table 3:** Biomechanical results of the finite element analysis

	ICCS	FNS	PCCP
Maximum femoral head displacements (mm)	5.0596	4.0276	2.7964
Maximum torsional displacements (mm) of fracture surfaces	3.45	2.3903	2.0058
Internal fixation maximum displacement (mm)	4.5446	3.0472	2.5585
Peak von Mises stress (MPa) of internal fixation	512.21	242.86	413.85

### Displacement of the internal fixation

The maximum displacement of internal fixation components occurred at the top of the screws, with values of 4.545 mm for ICCS, 3.047 mm for FNS, 2.556 mm for PCCP (Fig. 5g–i and Table 3).

### von Mises stress (VMS) of internal fixation

The VMS distributions and peak values for the three internal fixation models are shown in Fig. 5j–l and Table 3. In the ICCS group, the VMS was predominantly concentrated on the surface of the middle screws near the fracture line, with a uniform distribution along the direction of the screws. In the FNS group, the VMS concentration was noted at the junction of the sliding hip screw and antirotational screw, with a uniform distribution along the screw. In the PCCP group, the VMS was concentrated at the top hip screw near the fracture sites, with a uniform distribution along the top screw. Specifically, the peak VMS values of the internal fixation were 512.21 MPa for the ICCS, 242.86 MPa for FNS, and 413. 85 MPa for the PCCP. The maximum internal fixation stresses exerted by ICCS and PCCC were approximately 1.7–2.1 times higher than those of FNS. The maximum internal fixation stresses of both ICCS and PCCP were observed in the screws arranged at the top.

### Synthesized bone models

#### Displacements under loading

**Fig. 6:**
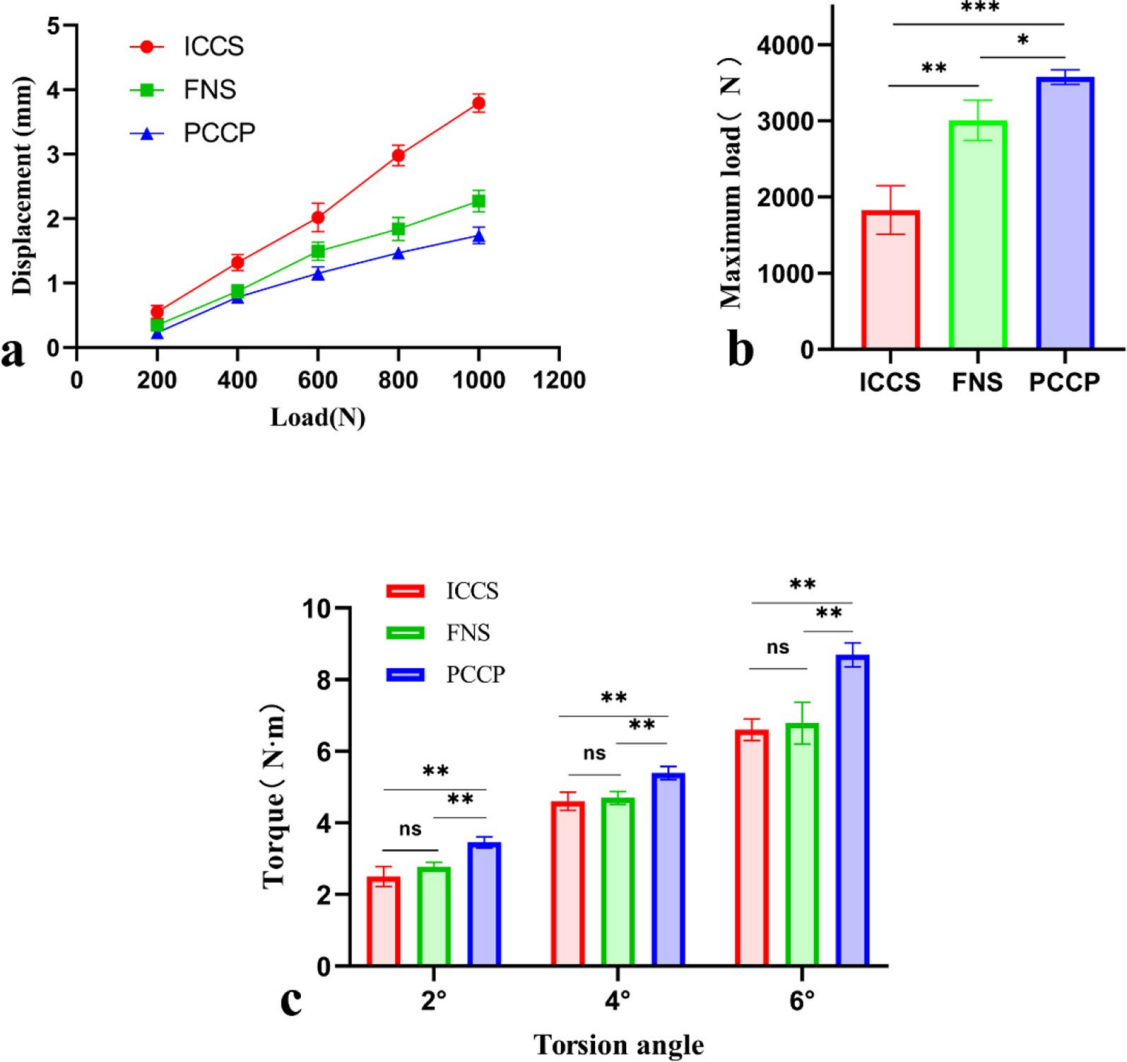
Biomechanical outcomes in the synthetic femur bone models. **a** the fracture displacements of the specimens under vertical loading. The loading force was increased from 200 to 1000 N on the femoral head in increments of 200 N. **b** The maximum vertical loads of specimens fixed by ICCS, FNS, and PCCP. The levels of vertical loads were increased horizontally until the fixation failed, at which point the value represented the ultimate load. **c** Torque values at different rotation angles. Torque forces were required to achieve rotation angles of 2°, 4°, and 6° in all groups. All data are presented as means ± standard deviations, *n* = 3 independent experiments. *P < 0.05, **P < 0.01, or ***P < 0.001 by ANOVA with LSD post-test

Under vertical loads of 200 N and 400 N, the displacements of specimens fixed with FNS and PCCP were significantly lower than those fixed with ICCS. However, no significance could be observed between FNS and PCCP. When vertical loads were increased to 600 N, 800 N, and 1000 N, the displacement of PCCP group was significantly less than that of the FNS group, which, in turn, was significantly lower than that of the ICCS group (Fig. 6a). The displacements of artificial bone models under various stress loads were detailed in Table 4**.**

**Table 4:** Biomechanical results of synthetic bone models under stress load and torsion loading tests (*n* = 3/each group).

		ICCS	FNS	PCCP	One-way ANOVA (*P* value)	LSD post hoc tests (*P* value)		
						ICCS versus FNS	ICCS versus PCCP	FNS versus PCCP
Displacements (mm) of specimens under loads (N)	200	0.55 ± 0.10	0.35 ± 0.07	0.23 ± 0.05	0.006	0.016	0.002	0.12
	400	1.32 ± 0.12	0.87 ± 0.1	0.78 ± 0.08	0.001	0.002	0.001	0.312
	600	2.02 ± 0.22	1.49 ± 0.14	1.15 ± 0.10	0.002	0.007	0.001	0.043
	800	2.98 ± 0.16	1.84 ± 0.18	1.47 ± 0.08	< 0.001	< 0.001	< 0.001	< 0.001
	1000	3.79 ± 0.14	2.27 ± 0.17	1.74 ± 0.13	< 0.001	< 0.001	< 0.001	< 0.001
Axial stiffness (N/mm)	–	244.86 ± 2.84	415.03 ± 27.10	529.98 ± 23.08	< 0.001	0.001	< 0.001	< 0.001
Failure loads (N)	–	1829.33 ± 319.21	3006.33 ± 214.36	3574.00 ± 96.97	< 0.001	0.001	< 0.001	0.03
Torque (N × m) at different rotation angles (degrees)	2	2.51 ± 0.28	2.78 ± 0.12	3.46 ± 0.15	0.003	0.144	0.001	0.006
	4	4.61 ± 0.25	4.70 ± 0.18	5.40 ± 0.18	0.007	0.594	0.003	0.006
	6	6.61 ± 0.30	6.80 ± 0.58	8.70 ± 0.33	0.002	0.616	0.001	0.002

Significant differences among these groups were evaluated using a one-way ANOVA, followed by an LSD post hoc multiple comparison tests. A *P* < 0.05 was considered statistically significant

With regards to axial stiffness, PCCP (529.98 ± 23.08 N/mm) was higher than FNS (415.03 ± 27.10 N/mm), which was higher than ICCS (244.86 ± 2.84 N/mm) (Table 4). The failure loads were observed to be higher for PCCP (3574.00 ± 96.97 N) compared with FNS (3006.33 ± 214.36 N), and FNS demonstrated a greater ultimate load capacity than ICCS (1829.33 ± 319.21 N) (Fig. 6b).

### Torque at different rotation angles

At rotation angles of 2°, 4°, and 6°, the torque required for the PCCP group was significantly higher than that for both the FNS group and ICCS group (*P* < 0.05), but there was no significant difference between the FNS group and ICCS group (*P* > 0.05) (Fig. 6c). The torque values at different rotation angles are presented in the Table 4. This indicated that PCCP required greater torque to achieve the same rotation angles compared with FNS and ICCS, suggesting that PCCP exhibited a smaller rotational deflection along the femoral neck axis than FNS and ICCS under identical torque conditions. In summary, PCCP and FNS demonstrated superior enhanced antirotational stability compared with ICCS, with PCCP slightly outperforming FNS.

## Discussion

The mechanical biological theory of fracture healing suggested that the mechanical environment surrounding the fracture site influenced the healing process [35, 36]. The reduction and fixation of the fracture is one of the essential aspects for fracture healing. In our study, the biomechanical outcomes of three internal fixations in the treatment of femoral neck fractures were explored using finite element analysis and verified using artificial bone models. The results indicated PCCP and FNS provided enhanced antirotational stability compared with ICCS, with PCCP slightly outperforming FNS. Notably, ICCS and PCCP exerted higher maximum internal fixation stresses than FNS, by approximately 1.7–2.1 times greater. The maximum internal fixation stresses of ICCS and PCCP occurred in the screws arranged at the top, suggesting a potential increased risk of breakage for screws positioned closer to the upper femoral neck. In the artificial bone models, PCCP exhibited superior axial stiffness and ultimate load capacity than ICCS. Moreover, PCCP demonstrates superior antirotation ability compared with both FNS and ICCS, though finite element analysis suggested similar antirotational performance for PCCP and FNS. 

The discrepancy between the finite element analysis and artificial bone model outcomes, particularly regarding antirotation capabilities, may be attributed to differences in measurement methods. The finite element analysis assessed displacement of the fracture surface under a uniform torque, whereas artificial bone models evaluated the torque required to achieve a specific rotation angle. 

Although there were some discrepancies between the simulated and experimental results, the trends in compressive strength and rotational resistance for these three groups (PCCP, FNS, and ICCS) were generally consistent between the finite element simulations and the experimental data. Some literature compared finite element analysis between FNS and ICCS. For example, the results of Teng et al. were consistent with our findings, indicating that the maximum stress of FNS was less than that of ICCS [37].

When the Pauwels angle was 65° and 70°, the maximum femoral displacement for FNS was slightly less than that for the CCS group [38, 39]. The finite element analysis provided valuable insights into the biomechanical behavior of internal fixation systems, which were useful for practical applications. We acknowledge that further refinement of the model could improve the accuracy of the simulation results. Future research might consider incorporating more detailed boundary conditions, such as the effects of surrounding muscles, ligaments, and the heterogeneous nature of the femur.

In recent years, some surgeons have applied recently introduced internal fixation devices, such as FNS and PCCP to the treatment of femoral neck fractures, which have significantly improved the therapeutic effects [18, 21].

The FNS is designed for dynamic fixation of femoral neck fractures, combining the advantages of angular stability and minimally invasive surgical techniques. A biomechanical study using cadaveric models had shown that FNS exhibits greater superior stability than ICCS in unstable Pauwels III femoral neck fracture models [23]. This biomechanical advantage may facilitate its clinical application. Surgical variations in the FNS used for the fixation of unstable femoral neck fractures (Pauwels type III) affected the stability outcomes. A finite element analysis revealed that positioning the bolt tip close to subchondral bone enhanced stability, whereas a lower blot implantation reduced it [40]. Certainly, further clinical data are required to validate these observations. Clinical studies have demonstrated that both FNS and PCCP fixation methods can provide high rates for femoral neck fracture healing and improve high hip scores in young adults. Compared with the ICCS group, the FNS group was associated with the reductions in operating time, exposure to fluoroscopic radiation, and the incidence of short-term complications, including femoral neck shortening and nonunion [18, 41].

PCCP is a dual-axis internal fixation device developed as an improvement on the DHS design. It consists of one lateral plate, two dynamically sliding femoral neck screws, and three locking screws. Its design was initially intended for the treatment of femoral trochanteric fractures. In 2007, Brandt first reported a case of PCCP applied to a Garden II femoral neck fracture with redisplacement after conservative treatment [42]. In 2010, Mukherjee et al. [43] reported a case of using PCCP to treat a femoral neck fracture with nonunion following ICCS fixation. They provided a new perspective for the treatment of femoral neck fractures. A biomechanical study [44] using synthetic and cadaver bones demonstrated that the axial compression and rotation resistance of PCCP is superior to DHS, which ensures early postoperative rehabilitation and load bearing, and the probability of internal fixation failure is small. Preliminary studies showed the application of PCCP in femoral neck fracture consistently leads to satisfactory fracture healing and functional recovery [19, 21]. Clinical studies have demonstrated that the Harris scores of femoral neck fracture patients in the PCCP group were significantly higher than those in the ICCS group at 6 and 12 months after surgery, suggesting that PCCP enhanced early functional recovery. However, PCCP did not have a significant impact on long-term outcomes [14]. In addition, the rates of internal fixation failure and bone nonunion in the PCCP group were significantly lower than those in the ICCS group, though the occurrence of avascular necrosis (AVN) showed no significant difference between the two groups [20]. In addition, PCCP for basal femoral neck fractures achieved a healing rate of up to 97% at 6-month follow-up based on imaging evidence [45].

There are both strengths and limitations in this study. One concern is whether our findings align with real-world clinical outcomes, given that finite element analysis relies on various assumptions to simplify models. For instance, bone models were assumed to be composed of continuous and isotropic elastic material, but in fact, actual human bones exhibited anisotropic and heterogeneous characteristics. Similarly, although artificial bone models are very close to the characteristics of human bone, they are not real human bone. Moreover, the joint capsule and ligament tissue around the hip joint were not considered in neither finite element analysis nor artificial bone models, which are critical for the stability and function of the hip joint.

Another limitation arises from material differences. The PCCP device was made of stainless steel, while the FNS and ICCS were made of titanium alloy. In artificial bone models, the difference in fixation material might introduce bias in the results. However, in our finite element analysis, assigning titanium alloy properties to the PCCP material did not significantly affect the trend of the results (data not shown). Patient-specific factors (e.g., age, sex, and osteoporosis) and surgical expertise might also impact the choice and success of fixation methods in clinical practice. These variables were not explicitly controlled for in our study and should be considered in future research. Furthermore, the varying costs associated with each fixation method were an important consideration. The cost effectiveness and availability of materials and implants might influence clinical decision-making, highlighting the need for a comprehensive cost–benefit analysis. Finally, our findings have not been confirmed by animal or clinical experiments. Nevertheless, the aim of this study is to identify trends rather than absolute measures, making the absence of further experimental confirmation rational.

The strength of our study is that the biomechanical properties of ICCS, FNS, and PCCP were first compared based on finite element analysis for unstable femoral neck fractures, with preliminary validation through synthesized bones. We believed that the insights from this study will offer valuable guidance for selecting internal fixation methods for Pauwels type III femoral neck fractures.

When compared with traditional ICCS, the internal fixation devices FNS and PCCP demonstrate superior resistance to compressive stress for Pauwels type III femoral neck fractures in both the finite element analysis and synthesized models. Both PCCP and FNS exhibit similar antirotation abilities in the finite element analysis, surpassing ICCS. However, in the synthetic bone model, FNS does not show a significant difference in antirotation capability compared with ICCS. Consequently, PCCP and FNS may be superior choices for managing Pauwels type III femoral neck fractures, but it should be noted that there is a risk of top screw breakage with PCCP and that FNS does not offer improved antirotation capability compared with ICCS. Additionally, adding a compression screw alongside FNS may potentially enhance its antirotational capabilities.

## Supplementary Information

Supplementary file 1.

## Data Availability

The datasets used and/or analyzed during the current study are available from the corresponding author on reasonable request.

## Abbreviations

ICCS: Inverted cannulated compression screw
FNS: Femoral neck system
PCCP: Percutaneous compression plate
VMS: von Mises stress
