# Supplementary material for: Biomechanical evaluation of percutaneous compression plate and femoral neck system in Pauwels type III femoral neck fractures
Source: J Orthop Traumatol. 2024 Nov 29;25:61. doi: 10.1186/s10195-024-00792-0 (PMC11607197; doi:10.1186/s10195-024-00792-0)
Supplement: Supplementary file 1 [file 10195_2024_792_MOESM1_ESM.docx]

**Supplementary Materials**

Supplementary Figs.1. The simplified modeling dimensions of ICCS in SolidWorks software.

Supplementary Figs.2. The simplified modeling dimensions of FNS in SolidWorks software.

Supplementary Figs.3. The simplified modeling dimensions of PCCP in SolidWorks software.

Supplementary Figs.4. The mesh diagram of the finite element analysis models in ANSYS software.

Supplementary Figs.5. The image of the internal fixation device (cannulated compression screws, FNS, PCCP).


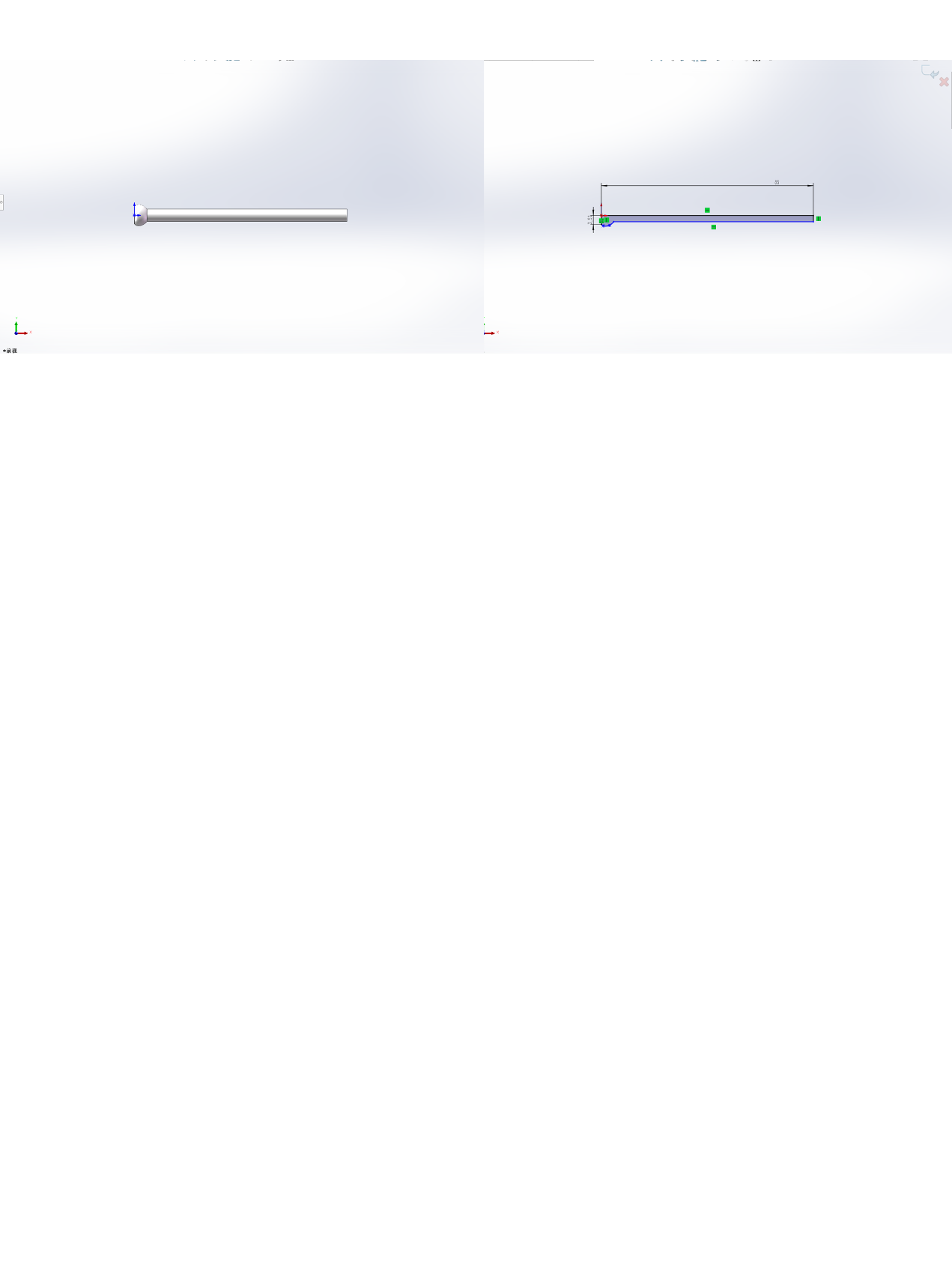


Supplementary Figs.1. The simplified modeling dimensions of ICCS in SolidWorks software.


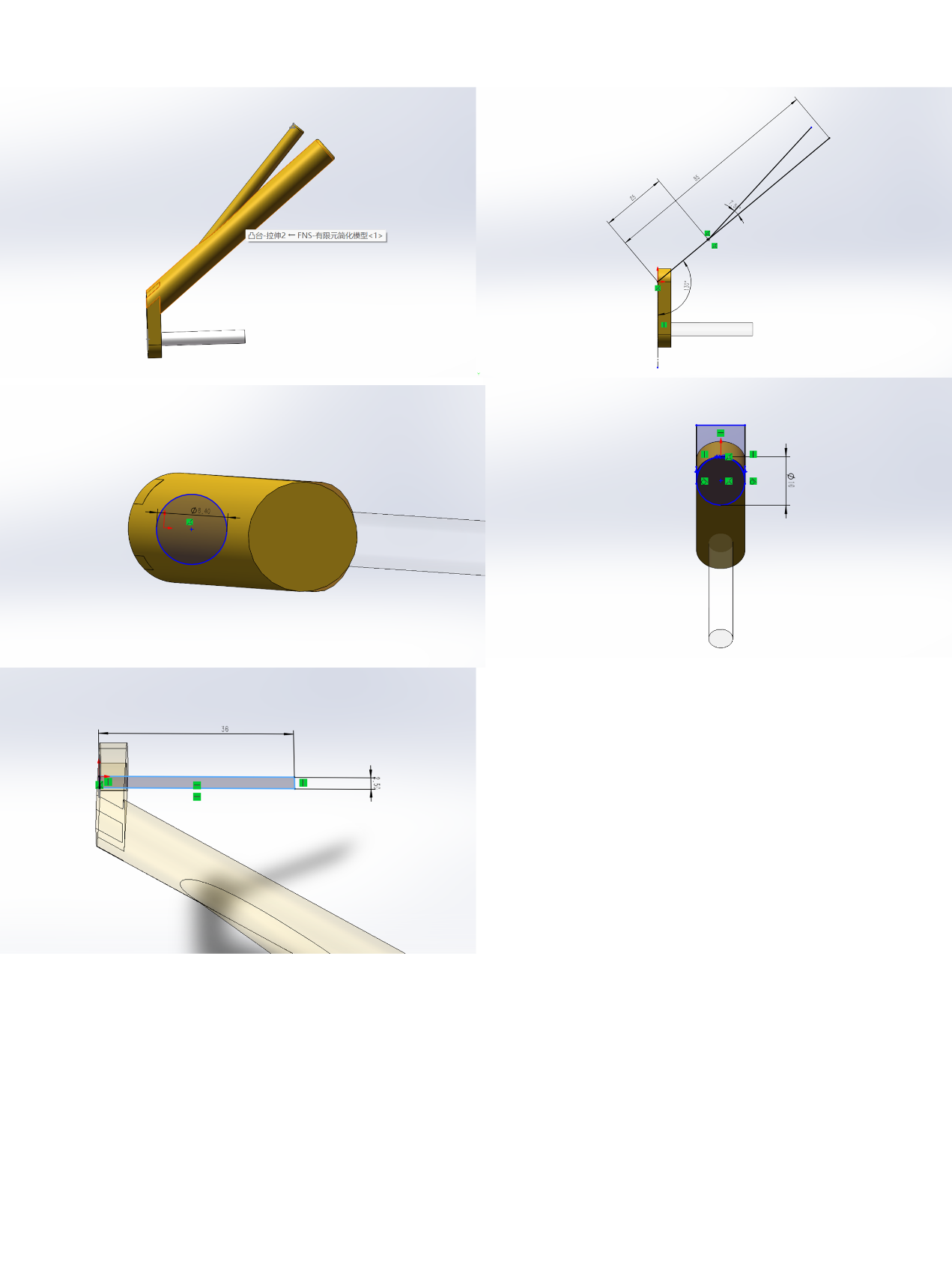


Supplementary Figs.2. The simplified modeling dimensions of FNS in SolidWorks software.


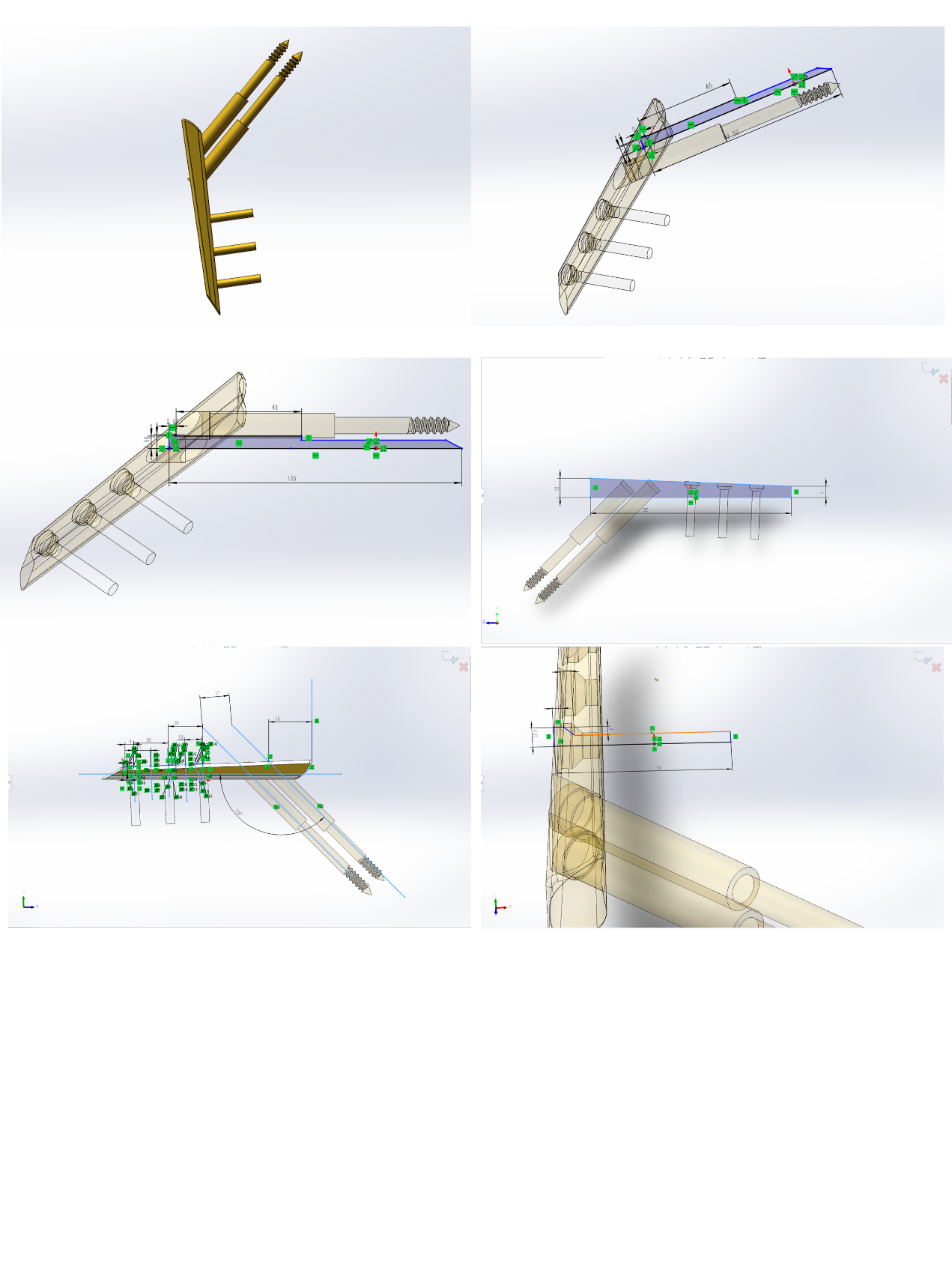


Supplementary Figs.3. The simplified modeling dimensions of PCCP in SolidWorks software.


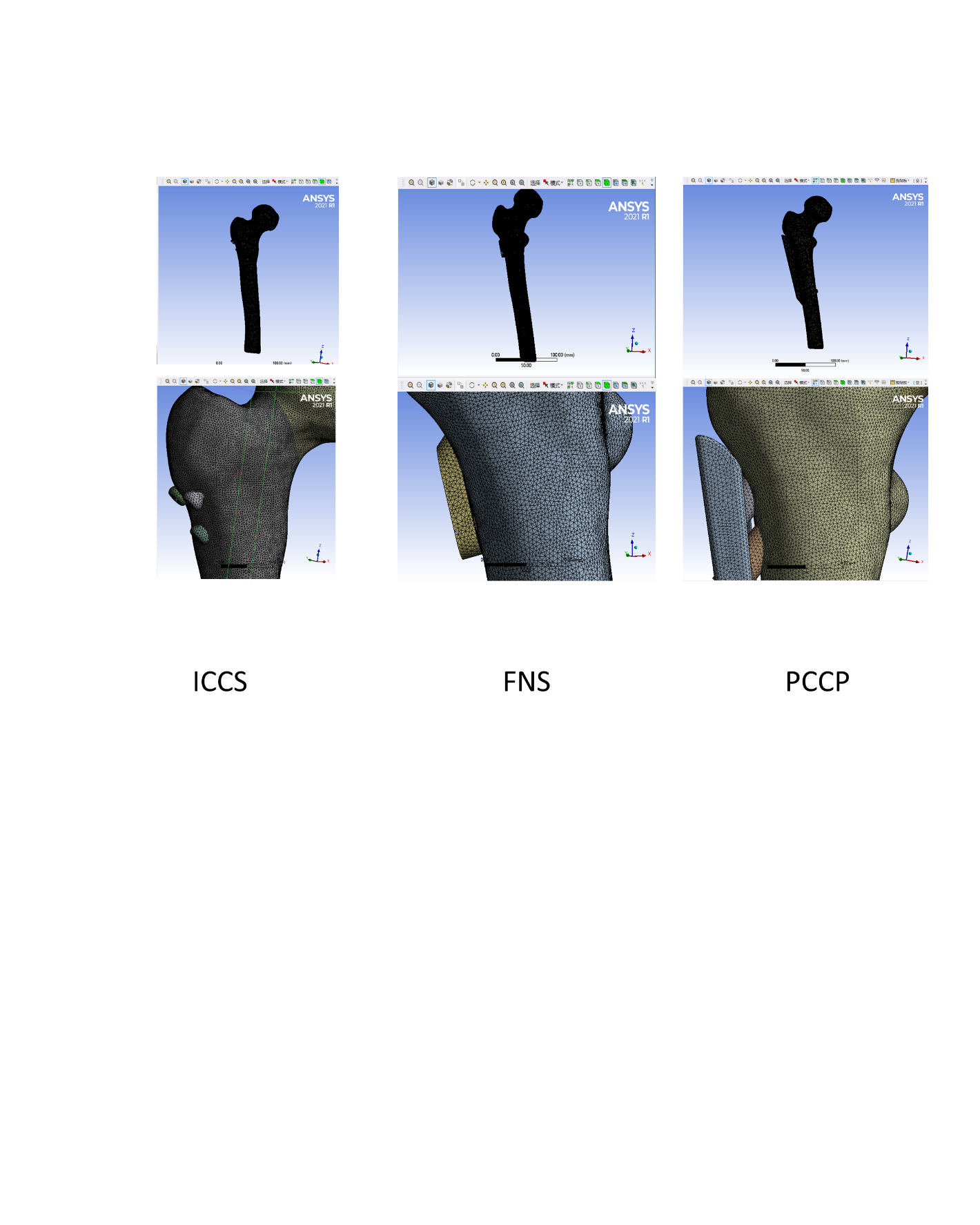


Supplementary Figs.4. The mesh diagram of the finite element analysis models in ANSYS software.


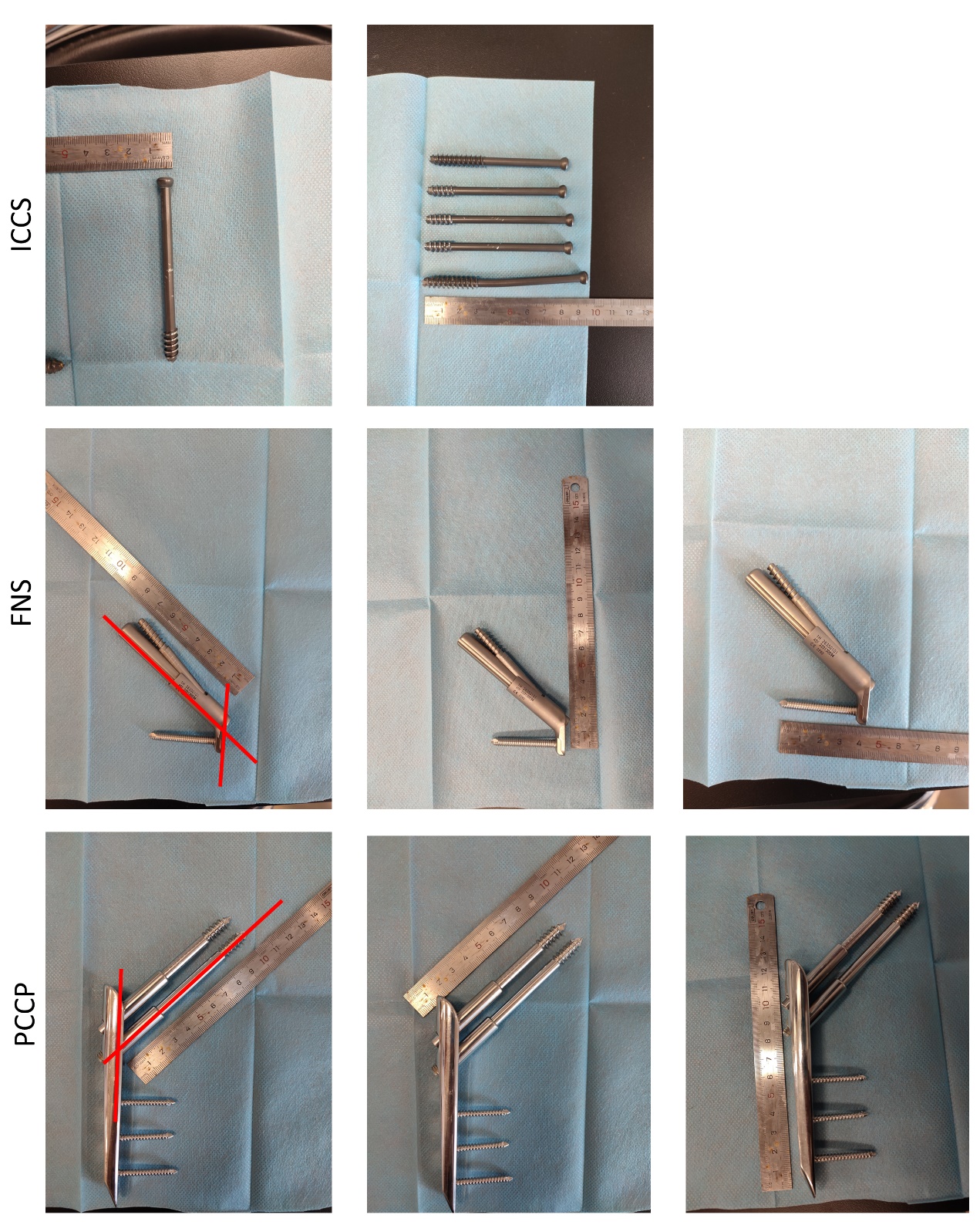


Supplementary Figs.5. The image of the internal fixation device (cannulated compression screws, FNS, PCCP).
